# The Global Changes of N6-methyldeoxyadenosine in Response to Low Temperature in *Arabidopsis thaliana* and Rice

**DOI:** 10.3390/plants12122373

**Published:** 2023-06-19

**Authors:** Fei Mao, Hairong Xie, Yucheng Shi, Shasha Jiang, Shuai Wang, Yufeng Wu

**Affiliations:** National Key Laboratory of Crop Genetics & Germplasm Enhancement and Utilization, Bioinformatics Center, Academy for Advanced Interdisciplinary Studies, Nanjing Agricultural University, Nanjing 210095, China; 2015201027@njau.edu.cn (F.M.); t2022087@njau.edu.cn (H.X.); 2021101130@stu.njau.edu.cn (Y.S.); 2021101131@stu.njau.edu.cn (S.J.)

**Keywords:** N6-methyldeoxyadenosine (6mA), low temperature, gene expression, orthologues gene, *Arabidopsis thaliana*, rice

## Abstract

N6-methyldeoxyadenosine (6mA) is a recently discovered DNA modification involved in regulating plant adaptation to abiotic stresses. However, the mechanisms and changes of 6mA under cold stress in plants are not yet fully understood. Here, we conducted a genome-wide analysis of 6mA and observed that 6mA peaks were predominantly present within the gene body regions under both normal and cold conditions. In addition, the global level of 6mA increased both in *Arabidopsis* and rice after the cold treatment. The genes that exhibited an up-methylation showed enrichment in various biological processes, whereas there was no significant enrichment observed among the down-methylated genes. The association analysis revealed a positive correlation between the 6mA level and the gene expression level. Joint analysis of the 6mA methylome and transcriptome of *Arabidopsis* and rice unraveled that fluctuations in 6mA levels caused by cold exposure were not correlated to changes in transcript levels. Furthermore, we discovered that orthologous genes modified by 6mA showed high expression levels; however, only a minor amount of differentially 6mA-methylated orthologous genes were shared between *Arabidopsis* and rice under low-temperature conditions. In conclusion, our study provides information on the role of 6mA in response to cold stress and reveals its potential for regulating the expression of stress-related genes.

## 1. Introduction

N6-methyldeoxyadenosine (6mA) is a prevalent DNA modification commonly found in lower organisms, such as bacteria, archaea, protozoa, and fungi. Its involvement in diverse biological processes includes gene transcription, DNA–protein interactions, DNA repair, and transposon insertion [1,2,3]. While 5mC is the primary DNA modification in higher eukaryotes, 6mA is present in trace amounts [1,4]. Advances in research have led to the increased recognition of 6mA’s features and roles in higher organisms. Recent studies using various techniques, such as liquid chromatography coupled with tandem mass spectrometry (LC-MS/MS), dot blot assays, 6mA-IP-seq, Single-molecule real-time (SMRT) sequencing, and Oxford Nanopore long-read sequencing, have revealed the widespread presence of 6mA in higher plant and animal species [5,6,7,8,9,10,11,12]. In *Chlamydomonas* and *Tetrahymena*, 6mA contributes to the localization of nucleosomes near the (transcription start site) TSS and appears to mark active genes [5,13]. In *Drosophila*, 6mA is enriched in transposons and affects their expression [7]. In *Arabidopsis* and rice, 6mA is widespread throughout the genome, and it is positively associated with the gene expression level when it marks in gene bodies [14,15,16]. Moreover, the roles of 6mA extend to various physiological processes, including fertilized egg development and human malignant glioma development [17,18,19]. Collectively, these findings indicate that 6mA plays versatile and essential roles in biological functions.

Stress-induced changes in DNA methylation and histone modifications have been shown to regulate stress responsive gene expression. For example, 5mC methylation of the *ALN* promoter is stimulated by cold, and this leads to the suppression of *ALN* expression and further promotion of seed dormancy [20]. Methylation of CHH in the *RhAG* promoter region regulates the expression of the *RhAG* gene, which impacts the development of rose petals under low temperatures [21]. Furthermore, recent studies have linked 6mA with environmental stress responses. The overall 6mA levels are significantly elevated upon stress in mouse brains, and 6mA dynamic changes were significantly associated to functional genes involved in learning, action, and neurogenesis [22]. For instance, heat stress response genes were marked by 6mA and H3K9me3 modification, resulting in high expression and extended lifespan in the progeny of *Caenorhabditis elegans* [23,24]. The 6mA level in mitochondrial DNA mediated by METTL14 was elevated under hypoxic stress in human cells [25]. These findings indicate a prevalent involvement of 6mA modification in response to environmental stresses, although the exact mechanisms underlying the regulation, especially in stress tolerance, are not fully understood.

Low temperature is a pervasive environmental stress that can negatively impact plant growth and development, ultimately reducing crop yields. Research over the past few decades has focused on the C-repeat binding factor/dehydration-responsive element-binding protein1 (CBF/DREB1)-dependent cold signaling pathway in *Arabidopsis*. The CBF proteins, CBF1–3, are transcription factors that activate the expression of *COR* (Cold-regulated) genes in response to cold by binding to conserved CRT/DRE motifs in their promoters [26,27,28]. In rice, several genes associated with chilling tolerance, including *Ctb1*, *qLTG3-1*, *COLD1*, *OsSAP16*, and *qCTS-9*, have been mapped and functionally characterized [29,30,31,32,33]. Despite the numerous advances in understanding responses to cold stress in plants, the role of 6mA in this process remains ambiguous.

Here, we profiled 6mA in the genomic DNA (gDNA) of *Arabidopsis* and rice under normal and cold conditions. We found 6mA was mostly located in gene body regions, and it positively correlated with gene expression. Cold treatment increased the overall level of 6mA methylation, and the differentially methylated genes (DMGs) exhibited enrichment in diverse biological processes. Joint analysis of the 6mA methylome and transcriptome of *Arabidopsis* and rice under normal and low temperatures revealed that 6mA-containing genes generally have higher expression levels than those without, and, while the overall level of 6mA increased under cold treatment, no correlation was found between its abundance and changes in expression level. Our results reveal that 6mA is a dynamic epigenetic mark in response to cold and participates in regulating of expression of stress responsive genes.

## 2. Results

### 2.1. The Distribution of 6mA and Its Association with Gene Expression in Arabidopsis and Rice under Normal Conditions

**Figure 1:**
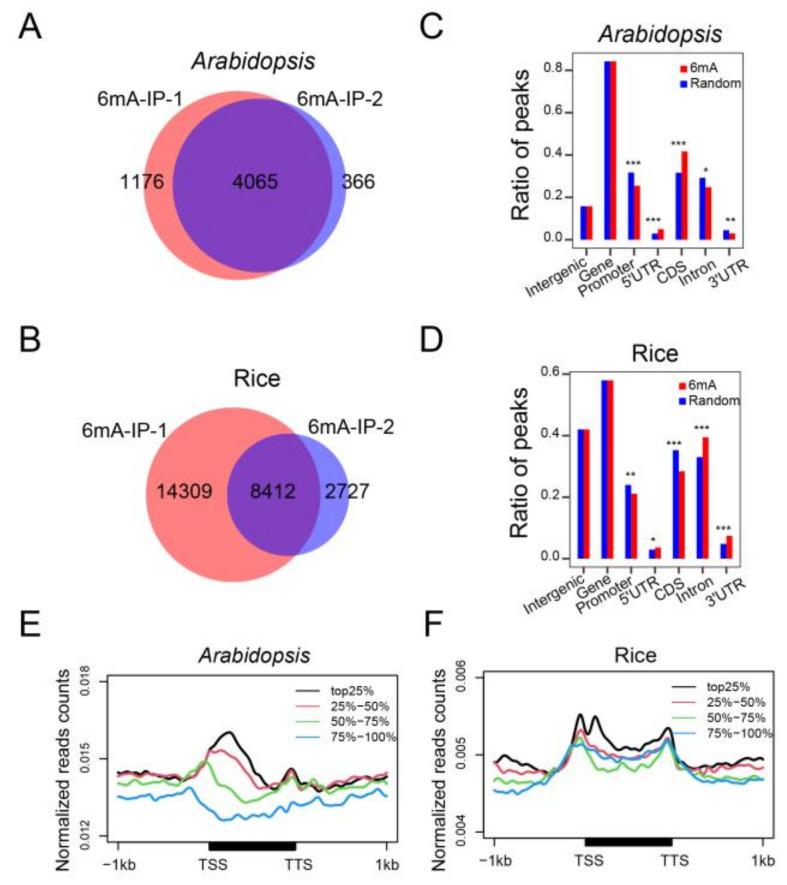
Genome-wide mapping and distribution of 6mA in *Arabidopsis* and rice under normal conditions. (**A**,**B**) Venn diagrams showing the overlapped 6mA peaks between two biological repeats in *Arabidopsis* (**A**) and rice (**B**) under normal conditions. (**C**,**D**) Distributions (expressed as percentages) of 6mA peaks found among intergenic regions, promoters (within 1 kb upstream of the TSS), and gene bodies. Gene bodies were further divided into 5′ and 3′ UTRs (untranslated regions), CDS (coding sequences), and introns. (* *p* < 0.05; ** *p* < 0.01; *** *p* < 0.001, Fisher’s exact test). (**E**,**F**) Metaplot showing that 6mA is positively correlated with gene expression levels in *Arabidopsis* (**E**) and rice (**F**). All genes were divided into 4 groups, from high to low (top 25%, 25–50%, 50–75%, and 75–100% based on FPKM) expression levels. TSS, transcription start site; TTS, transcription terminal site.

To investigate the distribution and association of the 6mA modification with gene expression in *Arabidopsis* and rice, we performed 6mA-IP (immunoprecipitation)-seq with two biological replicates for each species. We obtained more than 70 million unique mapped reads per 6mA-IP-seq sample for subsequent analysis (Table S1). High reproducibility of the sequence data was observed with high Pearson correlation coefficients (R > 0.97) in *Arabidopsis* and rice between the two biological replicates (Figure S1A,B). We identified 4065 high-confidence 6mA peaks in *Arabidopsis* (Figure 1A; Table S2) and 8412 high-confidence 6mA peaks in rice (Figure 1B, Table S3) after peak calling. To examine the epigenetic roles of 6mA, we investigated its location in the genome, including intergenic regions, promoter, gene bodies, and their subregions. A majority of the 6mA peaks were located in gene body regions in both *Arabidopsis* and rice. In *Arabidopsis*, 82% of the 6mA peaks were positioned in gene bodies, with half of the peaks occupying exons (Figure 1C). In rice, more than half of the peaks were located in gene bodies, especially in the intron regions (Figure 1D). These observations indicated a high enrichment of 6mA peaks on gene bodies in both species.

We also investigated the relationship between 6mA abundance and gene expression level. To this end, we carried out RNA-seq with three biological replicates to analyze the gene expression level. The correlation between the individual gene expression levels demonstrated high Pearson correlation coefficients (R > 0.88) (Figure S2A,B). We divided all the protein-coding genes in the genome into four classes based on their expression level: top 25%, 25–50%, 50–75%, and 75–100%. Plotting of 6mA abundance in these four classes revealed that strongly expressed genes had a higher occupancy of 6mA, particularly at the TSS region, than weakly expressed genes in both *Arabidopsis* and rice (Figure 1E,F). Thus, the 6mA modification is positively correlated with gene expression.

### 2.2. Genome-Wide Mapping of 6mA under Low Temperature

**Figure 2:**
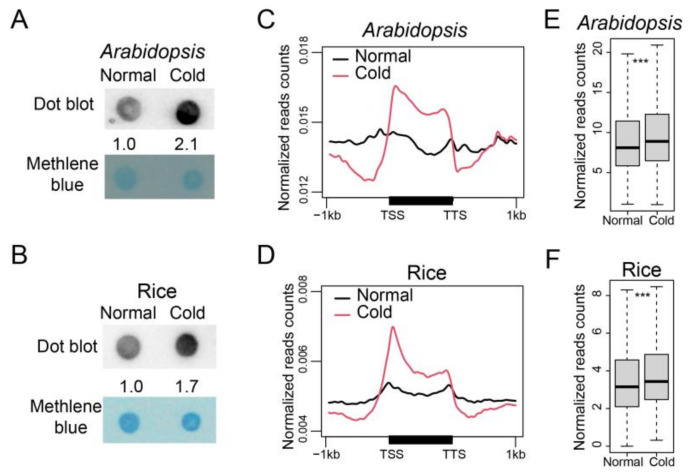
The overall 6mA level increased under cold. (**A**,**B**) 6mA dot-blot of genomic DNA samples from 3-week-old Col-0 plants (**A**) and 2-week-old rice seedlings (**B**) with 4 °C cold treatment for 24 h and 6 d, respectively. An amount of 200 ng of genomic DNA was loaded. Marked numbers indicate the relative amount in cold-treated samples compared to non-treated sample quantified by Image J. (**C**,**D**) 6mA profiles of *Arabidopsis* (**C**) and rice (**D**) under normal and cold conditions in gene regions. The 1 kb upstream and downstream flanking coding regions were aligned for all genes. TSS, transcription start site; TTS, transcription terminal site. (**E**,**F**) Box plot of the enrichment of 6mA peaks in *Arabidopsis* (**E**) and rice (**F**) under normal and cold conditions. The *p*-values were calculated for significant differences between two groups by the Mann–Whitney *U* test. ***: *p* < 0.001.

To explore whether 6mA responds to cold stress, we examined the overall 6mA methylation level after cold treatment (4 °C). We performed dot-blot assays for rosette leaves of 3-week-old *Arabidopsis* and 2-week-old rice seedlings. The 6mA signals were detected to have increased 2.1- and 1.7-fold, respectively, following a 24 h cold treatment in *Arabidopsis* and a 6 day cold treatment in rice (Figure 2A,B). We then profiled 6mA distribution for *Arabidopsis* and rice plants under cold conditions using 6mA-IP-seq. Two biological replicates were performed for each species, and high Pearson correlation coefficients (R > 0.99) were observed between the two biological replicates (Figure S3A,B). Peak calling led to the identification of 7557 and 19,013 high-confidence 6mA peaks in *Arabidopsis* and rice under cold conditions, respectively (Figure S3C,D, Tables S2 and S3). We further analyzed the distribution of 6mA methylation within each gene by plotting the read number ratio of m6A-IP-seq along the gene body region (from 1kb 5′ of TSS to 1 kb 3′ of TTS). In both *Arabidopsis* and rice, 6mA methylation showed a higher enrichment on the gene body region under cold conditions compared to normal conditions (Figure 2C,D). Box plot analysis confirmed a significant increase in the overall enrichment of 6mA peaks after cold treatment in both *Arabidopsis* and rice (Figure 2E,F). These results demonstrate that the genome-wide 6mA methylation level is increased under low temperature conditions.

### 2.3. Identification and Gene Ontology (GO) Analysis of Differentially Methylated Genes (DMGs) in Response to Cold Stress

**Figure 3:**
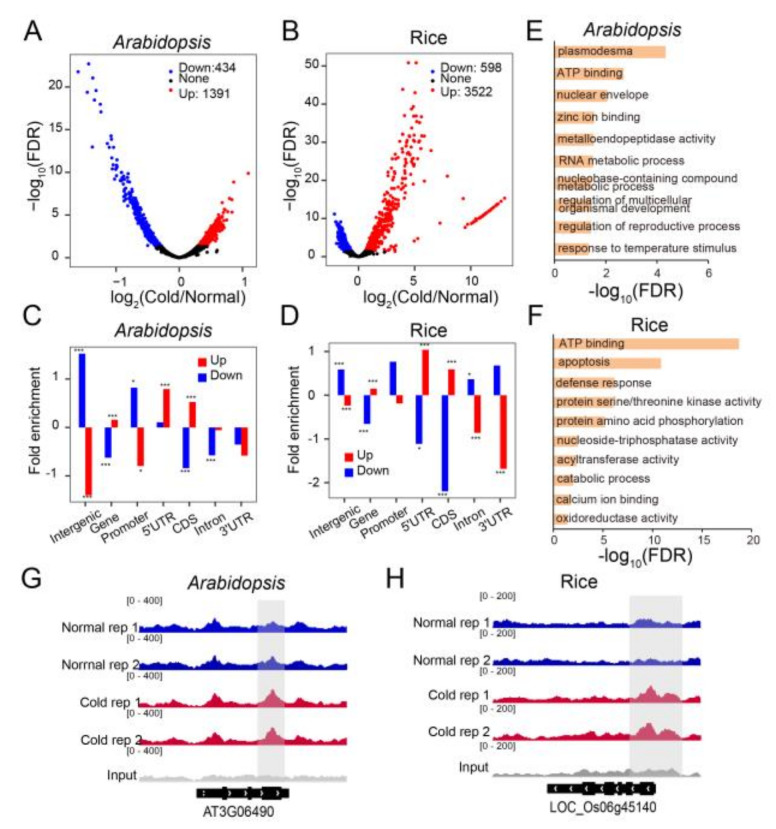
Identification and gene ontology (GO) analysis of DMGs in *Arabidopsis* and rice upon cold treatment. (**A**,**B**) Volcano plot showing the changes of significantly up- and down-regulated enrichment of 6mA peaks after cold treatment in *Arabidopsis* (**A**) and rice (**B**). Each dot represents 6mA-modified region under normal and cold conditions. Fold changes in 6mA reads (Cold/Normal) are indicated on the x-axis, and -log10 (FDR) of each 6mA-modified region is indicated on the y-axis. (**C**,**D**) Barplot showing the changes of 6mA enrichment among intergenic regions, promoters (within 1 kb upstream of the TSS), and gene bodies in *Arabidopsis* (**C**) and rice (**D**) after cold treatment. Gene bodies were further divided into 5′ and 3′ UTRs, CDS (coding sequence), and introns. (* *p* < 0.05; *** *p* < 0.001, Fisher’s exact test). (**E**,**F**), GO analysis of up-DMGs in *Arabidopsis* (**E**) and rice (**F**) after cold treatment. (**G**,**H**) Genomic visualization of 6mA-IP-seq for *MYB108* (AT3G06490) in *Arabidopsis*. (**G**) and *bZIP52* (LOC_Os06g45140) in rice (**H**) under normal and cold conditions.

In order to further examine 6mA alterations caused by chilling, we selected differentially methylated regions (DMRs) defined by a 1.5-fold or greater change in 6mA enrichment and with a significant test at q < 0.05. In *Arabidopsis*, we identified a total of 1391 up-regulated and 434 down-regulated differentially methylated regions (DMRs) (Figure 3A, Table S4), while in rice, we identified 3522 up-regulated and 598 down-regulated DMRs between cold and normal temperatures (Figure 3B, Table S5). We then analyzed the distribution of DMRs and found that down-regulated DMRs were primarily enriched in the intergenic and promoter regions, while up-regulated DMRs were mainly enriched in the coding sequence (CDS) and 5′ untranslated region (UTR) in both *Arabidopsis* and rice (Figure 3C,D). These up- and down-regulated DMRs corresponded to 1427 and 255 genes in *Arabidopsis*, which we defined as differentially methylated genes (DMGs), as shown in Table S4. The GO analysis of these DMGs revealed that up-DMGs were enriched in diverse terms such as plasmodesmata, ATP binding, response to temperature stimuli, and others (Figure 3E, Table S5). In rice, these up- and down-regulated DMRs corresponded to 2339 and 211 DMGs, respectively (Table S6). GO analysis of the up-regulated DMGs revealed enriched terms such as ATP binding, apoptosis, defense response, and others (Figure 3F, Table S7). No GO term was significantly enriched for the down-regulated DMRs in either *Arabidopsis* or rice. Furthermore, we discovered that the 6mA enrichment in key stress response genes increased after cold treatment. For instance, the *Arabidopsis* MYB family transcription factor *MYB108*/*BOS1* and the rice bZIP transcription factor *OsbZIP52/RISBZ5*, known for their involvement in abiotic stress regulation, displayed substantially increased 6mA levels under cold stress (Figure 3G,H). These findings suggest that 6mA plays a role in the regulation of diverse biological processes in *Arabidopsis* and rice.

### 2.4. The Effect of 6mA Modification on Gene Expression in Response to Low Temperatures

To investigate the role of 6mA modification in regulating gene expression in response to low temperatures, we initially analyzed the global association of 6mA on gene expression. Individual gene expression was analyzed using RNA-seq with three biological replicates of *Arabidopsis* and rice, where high Pearson correlation coefficients (R > 0.95) were observed (Figure S2A,B). To ensure the accuracy of our expression profiling data, we conducted a comparison with published RNA-seq data in *Arabidopsis* available from NCBI. However, we were unable to find comparable expression profiling data in rice at the same growth stage and under equivalent cold treatment conditions. As an alternative, we employed RNA-seq data obtained by Jabre et al. for their cold-treated (SRR10553430, SRR10553431, and SRR10553432) and control (SRR10553433, SRR10553434, and SRR10553435) samples [34]. By analyzing this data, we identified 11,604 differentially expressed genes (DEGs), which is slightly less than the 14,293 DEGs found in our study. Importantly, over 74% of the DEGs were common between these independent studies, providing strong support for the reliability and validity of our data (Figure S2C).

**Figure 4:**
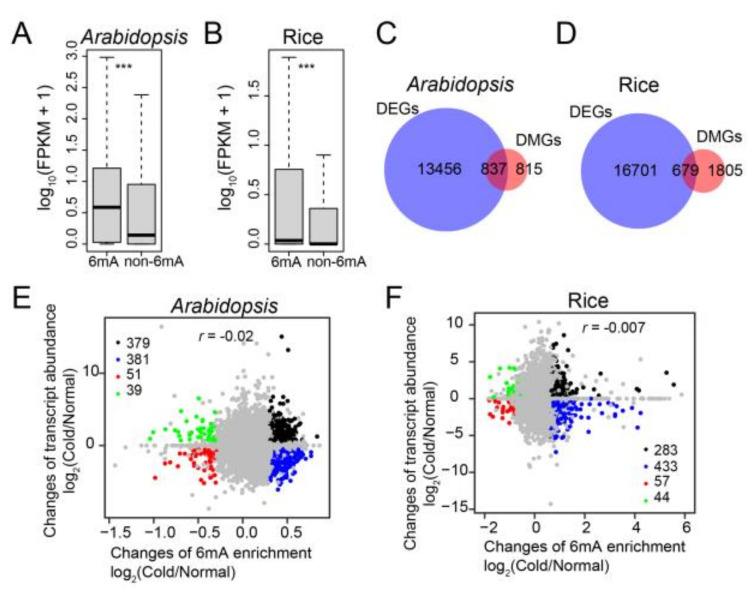
Association analysis of DMGs and DEGs in *Arabidopsis* and rice upon cold treatment. (**A**,**B**) Box plot of gene expression levels (based on FPKM values) for genes with or without 6mA peaks (6mA and non-6mA) in *Arabidopsis* (**A**) and rice (**B**) under normal and cold conditions. The *p* values were calculated for significant differences between the two groups by the Mann–Whitney *U* test. ***: *p* < 0.001. (**C**,**D**) Venn diagrams showing the overlaps between DMGs and DEGs in *Arabidopsis* (**C**) and rice (**D**) after cold treatment. (**E**,**F**) Correlation of fold changes between 6mA modification level and the expression of 6mA-containing genes in *Arabidopsis* (**E**) and rice (**F**) in response to low temperature.

The genes were subsequently divided into two classes, 6mA-containing genes and non-6mA-containing genes, to assess the association between 6mA modification and transcript abundance. Both *Arabidopsis* and rice showed that 6mA-containing genes had a higher average expression level than non-6mA-containing genes under cold conditions (Figure 4A,B). This indicates a positive role of 6mA modification in gene expression levels under low temperature.

We investigated whether the cold-induced alterations of 6mA modification affected changes in gene expression. In *Arabidopsis* and rice, we identified a total of 14,293 (6804 up/7489 down) and 17,380 (8364 up/9572 down) differentially expressed genes (DEGs), respectively, when comparing 4 °C to 22 °C (Figure S4A,B, Table S8). We observed that 50.7% and 27.3% of DMGs overlapped with DEGs in *Arabidopsis* and rice, respectively (Figure 4C,D). Furthermore, these overlapped genes were significantly enriched in the response to stimulus (Figure S4C,D, Table S9). However, the DMGs of 4 °C versus 22 °C exhibited an increased or decreased level of expression at 4 °C versus 22 °C (Figure 4E,F). In *Arabidopsis* and rice, the Pearson correlations between the changes in 6mA modification and gene expression levels were −0.02 and −0.007, respectively (Figure 4E,F). This suggests that the fold changes in gene expression induced by cold were not correlated with 6mA enrichment in both *Arabidopsis* and rice.

### 2.5. The Response of 6mA-Modified Orthologous Genes between Arabidopsis and Rice to Low Temperature

**Figure 5:**
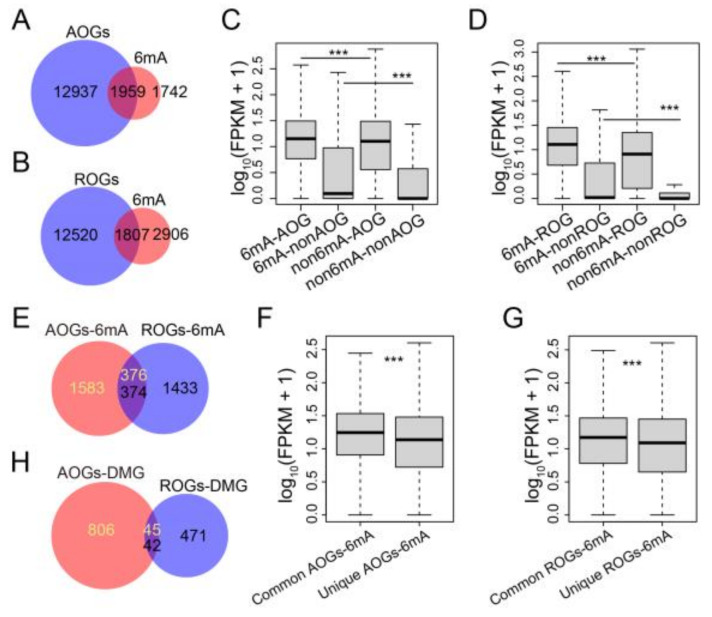
The response of 6mA-methylated orthologous genes between *Arabidopsis* and rice upon cold treatment. (**A**,**B**) Venn diagram showing the 6mA-methylated orthologous genes in *Arabidopsis* (**A**) and rice (**B**). (**C**,**D**) Box plot of gene expression for AOGs (**C**) and ROGs (**D**) with or without 6mA peaks (6mA and non-6mA). The *p* values were calculated for significant differences between the two groups by the Mann–Whitney *U* test. ***: *p* < 0.001. (**E**) Venn diagram showing the overlaps of 6mA-methylated orthologous genes between *Arabidopsis* and rice. (**F**,**G**) Box plot of gene expression for the common and unique 6mA-methylated orthologous genes in *Arabidopsis* (**F**) and rice (**G**). The *p* values were calculated for significant differences between the two groups by the Mann–Whitney *U* test. ***: *p* < 0.001. (**H**) Venn diagram showing the overlapped differentially 6mA-methylated orthologous genes between *Arabidopsis* and rice upon cold treatment.

To investigate the evolutionary conservation and functional significance of 6mA-modified orthologous genes between *Arabidopsis* and rice, we compared the sequence homology of genes in the two plants. We identified a total of 14,896 and 14,327 orthologs in *Arabidopsis* and rice, respectively, which we named *Arabidopsis* orthologous genes (AOGs) and rice orthologous genes (ROGs) (Figure S5A). Among these genes, 52.9% and 38.3% of 6mA-methylated genes overlapped with AOGs and ROGs, respectively, in *Arabidopsis* and rice (Figure 5A,B). Notably, the expression levels of 6mA-methylated orthologous genes were significantly higher than those of non-6mA-modified orthologous genes as well as 6mA-modified non-orthologous genes (Figure 5C,D). Furthermore, we identified about 370 common 6mA-methylated orthologous genes between *Arabidopsis* and rice, which exhibited higher expression levels than unique 6mA-methylated orthologous genes (Figure 5E–G). These results suggested that 6mA-methylated orthologous genes tend to be highly expressed. However, upon cold treatment, just over 40 differentially 6mA-methylated orthologous genes were shared between *Arabidopsis* and rice (Figure 5H), indicating that certain functions of 6mA-methylated orthologous genes may differ in response to cold. To investigate whether there are any common features among orthologous genes that exhibit changes in 6mA modifications between *Arabidopsis* and rice after cold treatment, we analyzed the trends in 6mA modification and expression level in response to low temperature. Our analysis revealed that over 70% of these genes display simultaneous upregulation in both *Arabidopsis* and rice, while only 26% exhibit similar alterations (both up or down) between the two species (Figure S5B,C). These findings are consistent with the global changes and relationship between 6mA modification and gene expression observed in both *Arabidopsis* and rice.

## 3. Discussion

6mA is a recently discovered DNA modification that plays a crucial role in numerous biological processes. Despite the low levels of 6mA in higher eukaryotes [9,35], recent research has confirmed its occurrence in animals and plants [10,14,15,16] and uncovered its function in various processes. This study focused on the biological implications of 6mA DNA modifications in *Arabidopsis* and rice under cold conditions. Our results revealed that there was a higher density of 6mA in gene bodies, which positively correlated with gene expression. Cold stress resulted in a higher abundance of 6mA, and up-DMGs exhibited involvement in various biological processes, but no GO term enrichment was observed for down-DMGs. Furthermore, we observed no correlation between changes in 6mA level and transcript level upon exposure to low temperature, indicating the intricate role that 6mA plays in regulating gene expression.

Under cold conditions, 6mA showed a significant increase in enrichment around the TSS region. The addition of a methyl group to the sixth position of adenine can potentially affect base-pairing energy and protein–DNA interactions and, in turn, influence transcription through the recruitment of transcription factors, RNA polymerases, histones, or other components within the surrounding chromatin context. Therefore, alterations in the position or density of 6mA in response to cold can potentially impact chromatin accessibility, nucleosome positioning, and gene expression level. Notably, other histone modifications, including histone acetylation (H3K9ac, H3K18ac, and H3K23ac) and histone methylation (H3K4me3 and H3K36me3), were also found to be present near the TSS [36,37,38,39], indicating that changes in 6mA due to low temperature likely interact with other histone modifications to modulate gene expression. Previous studies have demonstrated intricate interactions between DNA methylation and histone methylation. For example, past research has shown 5mC methylation to control histone H3K9 methylation, and a mutual enhancement between H3K9me2 and 5mC methylation has been observed in *Arabidopsis* [40,41,42]. The mechanism behind the association between 6mA and other epigenetic marks should be addressed in future research.

Previous studies have shown that overall changes in 5mC under abiotic stress in *Arabidopsis* and rice do not correlate with changes in gene expression [43]; however, 5mC is known to substantially modulate the expression of some stress responsive genes [20,44,45,46]. In our study, we found the changes of 6mA enrichment were also not correlated with alterations of gene expression after cold shift. GO analysis of up-DMGs revealed enrichment in diverse processes including temperature stimulus, and some important stress response genes demonstrated changes in 6mA level, indicating that 6mA potentially participates in the regulation of stress response gene expression. In this study, it was observed that the 6mA enrichment in *qLTG3-1*, which is a significant quantitative trait locus responsible for controlling low-temperature germinability in rice [29], increased when subjected to cold stress. Further research is necessary to investigate the significance of 6mA in adapting to environmental cues through the regulation of gene expression.

The distribution of 6mA and its association with transcription appear to differ between animals. For instance, 6mA sites are evenly distributed throughout the genome in worms [6], but in flies, they are enriched in transposable elements and correlate with transposon expression [7]. However, in this study, we found that the genomic distribution and association of 6mA with transcription are conserved between *Arabidopsis* and rice. The 6mA peaks were predominantly present within gene body regions, and the density of the 6mA peaks positively correlated with gene expression levels in both species. Additionally, we observed that 52.9% and 38.3% of 6mA-methylated genes overlapped with AOGs and ROGs in *Arabidopsis* and rice, respectively (Figure 5A,B). Moreover, common 6mA-methylated orthologous genes between *Arabidopsis* and rice exhibited higher expression levels than unique 6mA-methylated orthologous genes (Figure 5E–G). The evolution of 6mA is an interesting topic that requires further investigation.

In conclusion, this study found that the overall 6mA levels increase under low temperatures and analyzed 6mA-modified regions under both normal and cold conditions. The changes in 6mA modification due to low temperature were primarily distributed in gene body regions and displayed differential correlations with gene expression levels. The DMGs were enriched in various biological processes, including stress response, and several key stress response genes revealed differential methylation levels of 6mA following cold treatment. The discovery of the roles of 6mA epi-modification in adjusting to cold temperatures not only enhances our understanding of environmental adaptation but also broadens the options available for the generation of stress-tolerant plants.

## 4. Materials and Methods

### 4.1. Plant Materials and Growth Conditions

*Arabidopsis thaliana* Col-0 was grown in a growth chamber at 22 °C under long day conditions (16 h light/8 h dark) with a light intensity of 100 µmol m^−2^ s^−1^. Upon cold treatment, 3-week-old plants were transferred to 4 °C for 24 h. Rice (*Oryza sativa*, *Nipponbare*) was grown in a growth chamber at 28 °C under long-day conditions (16 h light/8 h dark) with a light intensity of 200 µmol m^−2^ s^−1^ for 2 weeks. For cold treatment, 2-week-old seedlings were transferred to 4 °C for 6 days.

### 4.2. Isolation of gDNA and 6mA-IP-Seq

gDNA of *Arabidopsis* and rice were extracted by using a DNAsecure Plant Kit (TIANGEN, Cat. DP320-03, Beijing, China) according to its procedures. Isolated gDNA was sonicated into 200–400 bp with Biorupter UCD-600, followed by end repair, 3′-adenylation, and adaptor ligation according to NEB Next Ultra II DNA Library Prep Kit for Illumina (E764S, NEB, Ipswich, MA, USA). The ligated DNA was denatured at 95 °C for 10 min and chilled on ice for 10 min. 10 µL of denatured DNA was saved as input. An amount of 1µg DNA was incubated with 3 µg of anti-m6A antibody (202-003, Synaptic Systems, Göttingen, Germany) at 4 °C for 6 h in 1 × IP buffer (10 mM Tris-HCl PH 7.4, 150 mM NaCl, 0.1% IGEPAL CA-630). Dynabeads Protein A (10001D, Invitrogen, Waltham, MA, USA) was washed twice with 1 × IP buffer and pre-blocked in 0.5 mL 1 × IP buffer with 20 µg/µL BSA for 2 h at 4 °C. After pre-blocked beads were washed twice with 1 × IP buffer, the DNA–antibody mixture was added to beads, rotating overnight at 4 °C. The beads were washed 4 times with 1 × IP buffer. Methylated DNA was eluted twice by 100 μL elution buffer containing N6-methyladenosine 5′-monophosphatesodium salt (M2780, Sigma, St. Louis, MO, USA) at 4 °C for 1 h. Eluted DNA was combined, then added to 20 μL of 3 M NaOAc (PH 5.3), 500 μL ethanol, and 0.5 μL glycogen. The mixture was frozen at −80 °C overnight and centrifuged. The precipitated DNA was dissolved in 20 μL ddH_2_O, followed by 8–10 cycles PCR amplification. The PCR product was purified by VAHTS DNA clean beads (N411-01, Vazyme, Nanjing, China). Sequencing was conducted by the Illumina NovaSeq6000 platform with 150-bp paired-end reads at Jiangbei New Area Biopharmaceutical Public Service Platform Co., Ltd. (Nanjing, China).

### 4.3. RNA Extraction and RNA-Seq

Total RNA of *Arabidopsis* and rice were isolated with TRIzol (15596026, Thermo Fisher, Waltham, MA, USA). Ribosomal RNA was removed by using an mRNA Miniprep Kit (Sigma, MRN10). An NEB Next Ultra II RNA Library Prep Kit for Illumina (NEB, E7770S) was used to construct the library, and sequencing was performed on an Illumina HiSeq 2000 platform with 100-bp paired-end reads at Jiangbei New Area Biopharmaceutical Public Service Platform Co., Ltd. (Nanjing, China).

### 4.4. Data Analyses

The reads of raw data were trimmed according to adapt sequence using cutadapt (v1.1) [47]. Then, the clean data were mapped to *Arabidopsis* genome (TAIR10) or rice genome (MSU7.0) using bowtie [48]; the parameter of maximum insert size was reset to 1000. Only unique reads were used to detect 6mA enrichment region by SCIER (v1.1) [49] (using parameters redundancy threshold = 1 window size (bp) = 200 fragment size = 100 effective genome fraction = 0.74 gap size (bp) = 200 FDR = 0.01). Overlapping peaks (at least 1 bp overlap) between two biological replicates of a sample were retained for next analyses. DESeq2 (1.24.0) [50] was used to compare all 6mA region from normal and cold stress with the consideration of sample variation. Differential regions with *p* value < 0.05 (false discovery rate < 0.05) were considered significant (DMRs).

The sequencing data were mapped to the *Arabidopsis* genome with matching annotation by tophat (v2.1.1) [51] using default parameters. The expression value (FPKM) of each gene was calculated with cufflinks (v2.1.1) [51], and cuffdiff (v2.1.1) [51] was used to calculate the fold change of gene expression and FDR. Differential expression genes (DEGs) with false discovery rate < 0.05 were considered significant.

The agriGO v2.0 [52] was used for GO analysis. The GO terms with FDR ≤ 0.05 were considered to be enriched. For GO terms with a hierarchical relationship, the child GO terms were kept and the parent terms were removed to avoid redundancy in this study. Orthofinder (v2.3.12) [53] was run to search homologous genes between rice and *Arabidopsis thaliana*. 

## Data Availability

All sequencing data generated from this study have been deposited into the NCBI GEO database under the accession number GSE230463 (Review token: efajkagklduppkt).

## Supplementary Materials

The following supporting information can be downloaded at https://www.mdpi.com/article/10.3390/plants12122373/s1. Figure S1. Pearson correlation analysis of 6mA-IP-seq read counts in *Arabidopsis* and rice under normal conditions; Figure S2. Pearson correlation analysis of RNA-seq data in *Arabidopsis* and rice under normal and cold conditions; Figure S3. Pearson correlation analysis of 6mA-IP-seq read counts in *Arabidopsis* and rice under cold conditions; Figure S4. The DEGs identified in *Arabidopsis* and rice after cold treatment; Figure S5. Characteristics of overlapping orthologous genes displaying changes in 6mA modifications between *Arabidopsis* and rice following exposure to cold treatment; Table S1. A summary of 4acC-IP-seq and RNA-seq information; Table S2. Identification of 6mA peaks under normal and cold conditions in *Arabidopsis*; Table S3. Identification of 6mA peaks under normal and cold conditions in rice; Table S4. Identification of DMPs and DMGs upon cold treatment in *Arabidopsis*; Table S5. GO analysis of up-DMGs in *Arabidopsis* after cold treatment; Table S6. Identification of DMPs and DMGs upon cold treatment in rice; Table S7. GO analysis of up-DMGs in rice after cold treatment; Table S8. The DEGs identified in *Arabidopsis* and rice after cold treatment; Table S9. GO analysis of the overlapped genes between DMGs and DEGs in *Arabidopsis* and rice upon cold treatment.
