# Supplementary material for: The Global Changes of N6-methyldeoxyadenosine in Response to Low Temperature in Arabidopsis thaliana and Rice
Source: Plants (Basel). 2023 Jun 19;12(12):2373. doi: 10.3390/plants12122373 (PMC10305114; doi:10.3390/plants12122373)
Supplement: Supplementary file 1 [file plants-12-02373-s001.zip › Figure S1-5.pdf]

Figure S1

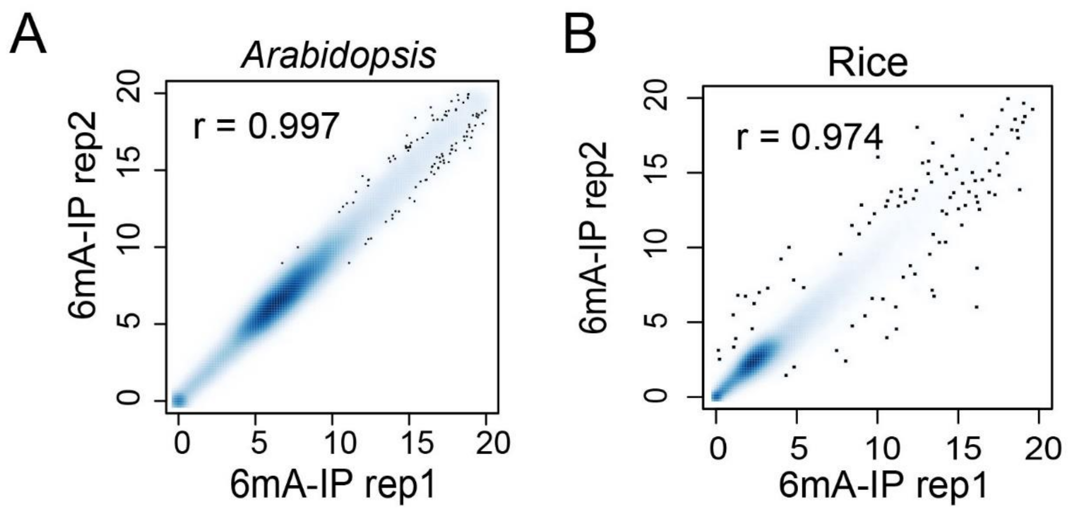

Figure S1. Pearson correlation analysis of 6mA-IP-seq read counts in Arabidopsis and rice under normal condition. (A) and (B) Pearson correlation analysis of 6mA-IP-seq read counts in Arabidopsis (A) and rice (B) between two biological replicates under normal condition. Rep, Replicate.

Figure S2

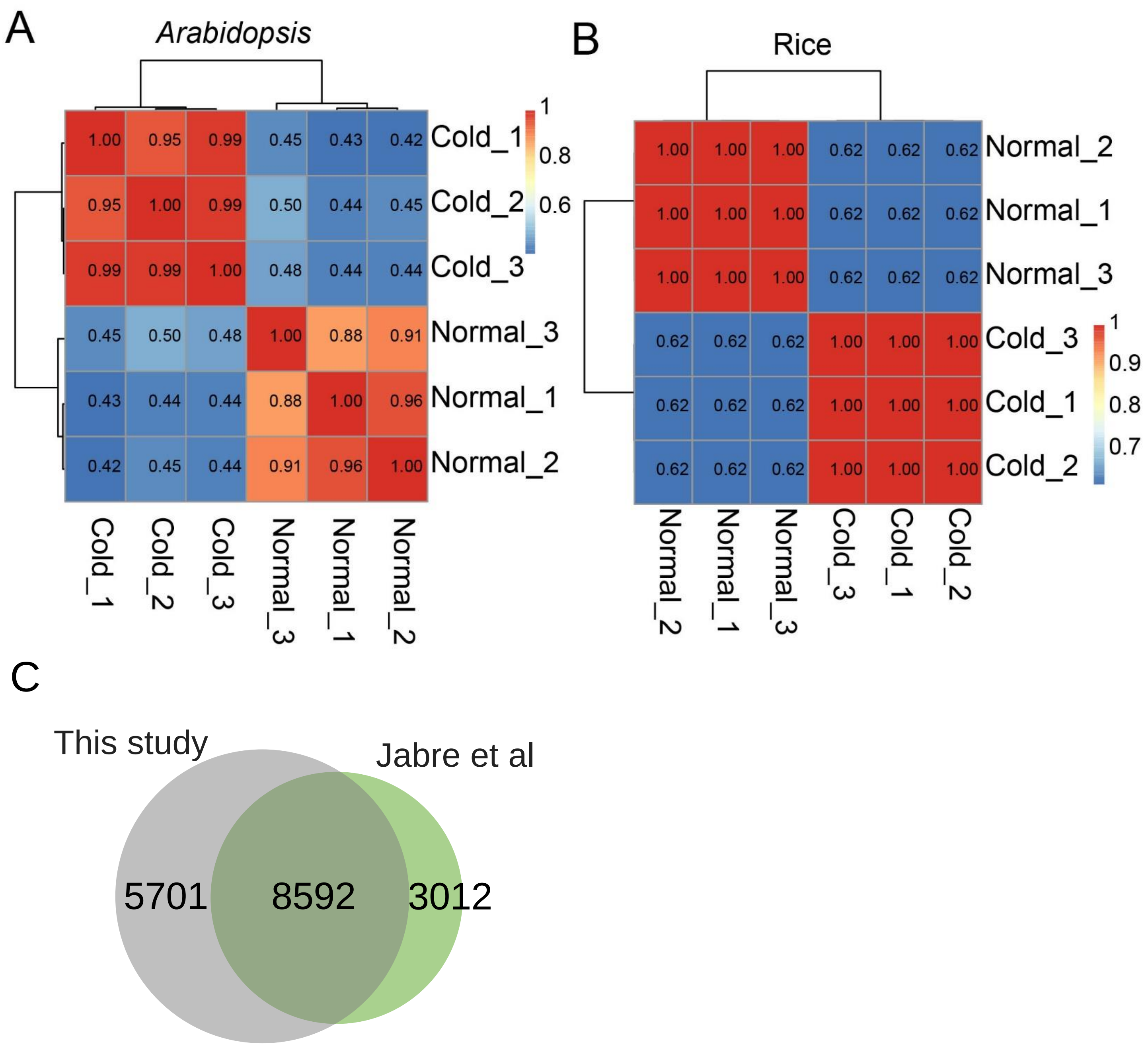

Figure S2. Pearson correlation analysis correlation analysis of RNA-seq data in *Arabidopsis* and rice under normal and cold condition. (A) and (B) Pearson correlation analysis of RNA-seq read counts in Arabidopsis (A) and rice (B) between three biological replicates. RNA-seq experiments were performed with three independent biological replicates from 3-week-old Arabidopsis and 2-week-old rice seedlings under normal and cold conditions. Rep, Replicate. (C) The overlaps of DEGs identified in this study with Jabre et al published data.

Figure S3

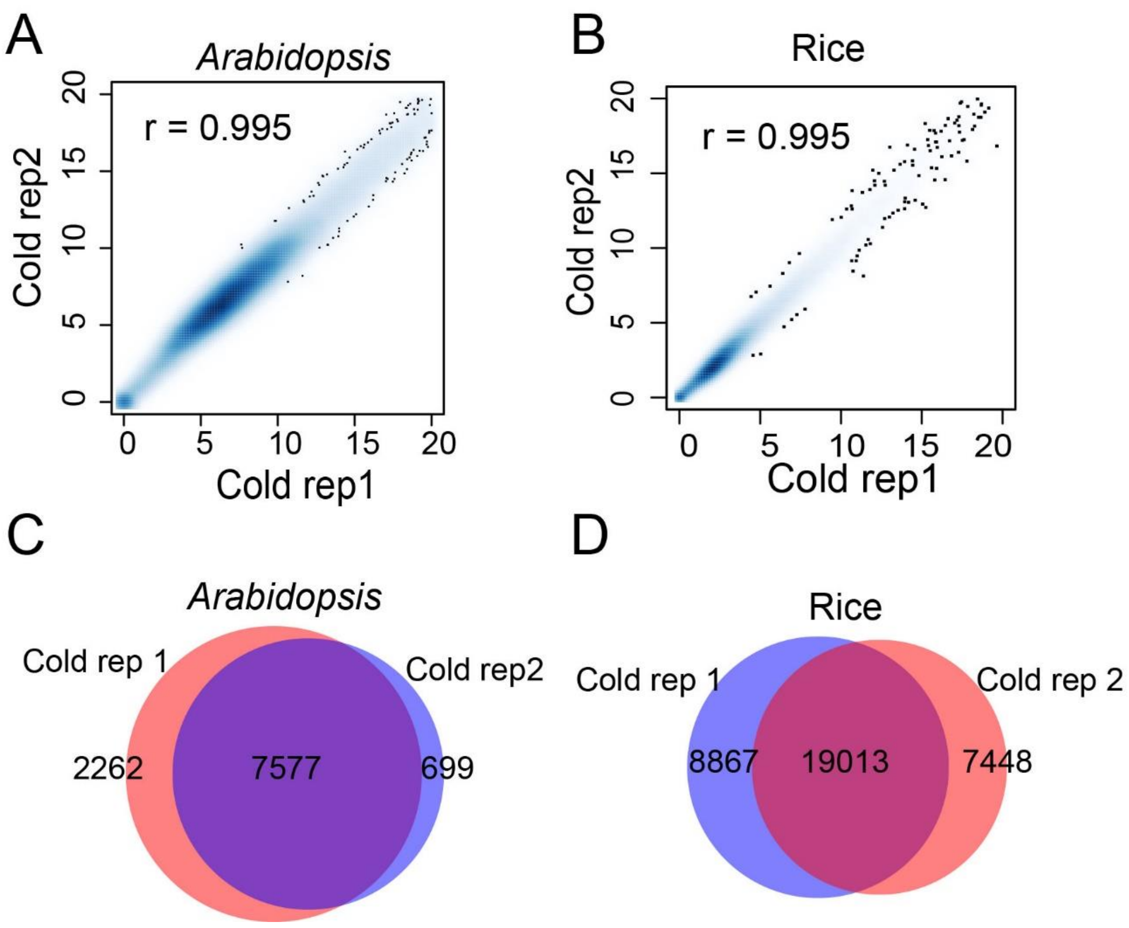

Figure S3. Pearson correlation analysis of 6mA-IP-seq read counts in Arabidopsis and rice under cold condition. (A) and (B) Pearson correlation analysis of 6mA-IP-seq read counts in Arabidopsis (A) and rice (B) between two biological replicates under cold condition. Rep, Replicate. (C) and (D) Venn diagrams showing the overlapped 6mA peaks between two biological repeats in Arabidopsis (C) and rice (D) under cold condition.

Figure S4

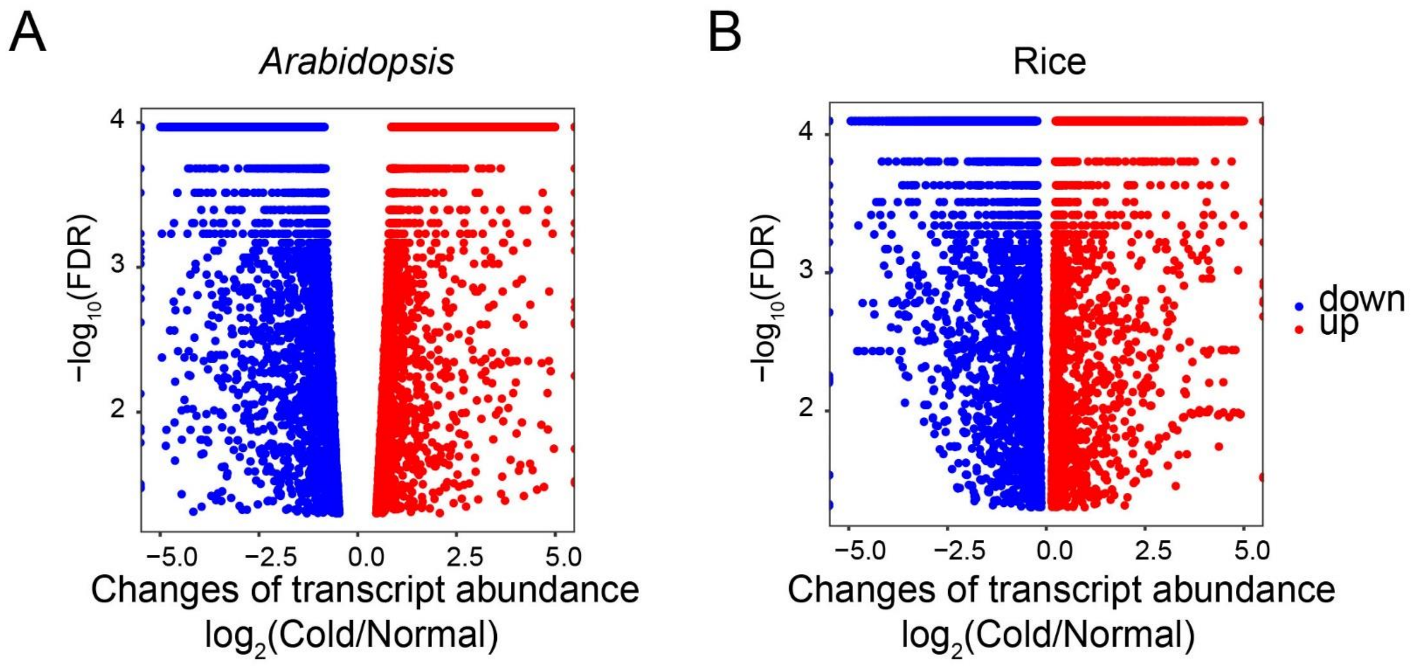

Figure S4. The DEGs in Arabidopsis and rice after cold treatment. (A) and (B) Volcano plot showing the changes of significantly up- and down-regulated expression of genes after cold treatment in Arabidopsis (A) and rice (B) .

Figure S5

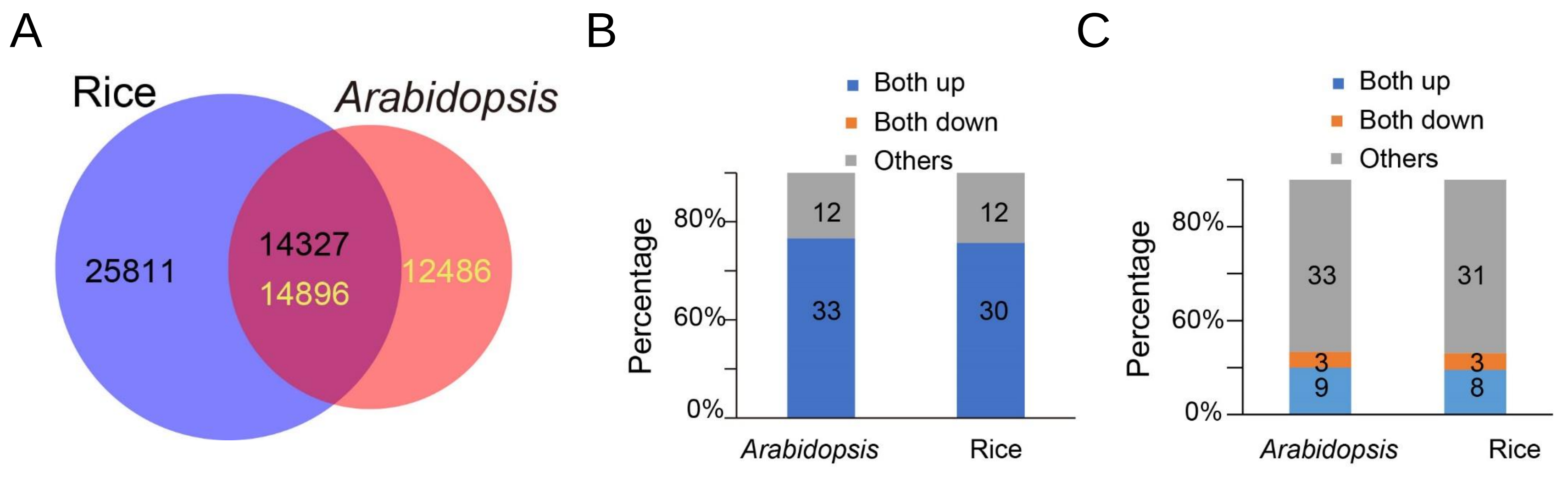

Figure S5. Characteristics of overlapping orthologous genes displaying changes in 6mA modifications between Arabidopsis and rice following exposure to cold treatment. (A) Venn diagram showing the orthologous genes between Arabidopsis and rice. (B) Percentage of genes showing analogous changes in 6mA modification levels in both Arabidopsis and rice. (C) Percentage of genes exhibiting analogous changes in expression levels in both Arabidopsis and rice. "Both up" indicates increased 6mA modification (B) or expression level (C) in both Arabidopsis and rice, while "Both down" indicates decreased 6mA modification (B) or expression level (C) in both Arabidopsis and rice. "Others" indicates that the genes display inconsistent changes in 6mA modification (B) or expression level (C) between Arabidopsis and rice.
